# Enhanced wetting of Cu on ZnO by migration of subsurface oxygen vacancies

**DOI:** 10.1038/ncomms9845

**Published:** 2015-11-16

**Authors:** Igor Beinik, Matti Hellström, Thomas N. Jensen, Peter Broqvist, Jeppe V. Lauritsen

**Affiliations:** 1 Interdisciplinary Nanoscience Center (iNANO), Aarhus University, Aarhus DK-8000, Denmark; 2 Department of Chemistry—Ångström, Uppsala University, Box 538, Uppsala SE-75121, Sweden

## Abstract

Metal adhesion on metal oxides is strongly controlled by the oxide surface structure and composition, but lack of control over the surface conditions often limits the possibilities to exploit this in opto- and micro-electronics applications and heterogeneous catalysis where nanostructural control is of utmost importance. The Cu/ZnO system is among the most investigated of such systems in model studies, but the presence of subsurface ZnO defects and their important role for adhesion on ZnO have been unappreciated so far. Here we reveal that the surface-directed migration of subsurface defects affects the Cu adhesion on polar ZnO(0001) in the technologically interesting temperature range up to 550 K. This leads to enhanced adhesion and ultimately complete wetting of ZnO(0001) by a Cu overlayer. On the basis of our experimental and computational results we demonstrate a mechanism which implies that defect concentrations in the bulk are an important, and possibly controllable, parameter for the metal-on-oxide growth.

## Abstract (web summary)

Comprehensive elucidation of metal-support interactions is important for controlling and improving their performances in a range of pertinent technologies. Here, the authors reveal how subsurface defects influence the adhesion and wetting of a metal on the surface of a metal oxide.

## Graphical abstract

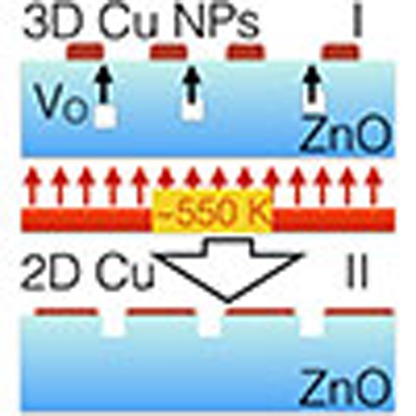

The electronic and structural properties at the interface between a metal and a semiconducting or insulating surface are of key relevance to semiconductor devices, oxide-based sensors and opto- and micro-electronics. The interaction between a metal nanoparticle (NP) and a metal oxide support is also a key parameter in catalysis where it receives a considerable amount of attention since the stability and activity of the catalyst relies on good control of the metal NP adhesion to its support. Consequently, fundamental insight into interface engineering is of high priority for such systems. The Cu/ZnO metal-support system is currently one of the most studied owing to its attractive catalytic properties^1^. Heterogeneous catalysts, combining Cu/ZnO with inert aluminium oxide, are extensively used at the industrial scale for selective methanol synthesis, the water gas shift reaction, and can be used for on-board hydrocarbon conversion in fuel cells. It has recently been demonstrated that the closely related CuO/ZnO and Cu_2_O/ZnO systems constitute highly active photocatalysts for water reduction^2^,^3^. Copper is also an important dopant in ZnO^4^, which can be used to control its electrical, optical and sensing properties.

Recent studies on Cu/ZnO-based catalysts demonstrated that the presence of defects on the surfaces of ZnO crystallites may have a pronounced effect on the activity of the catalyst^5^,^6^. The elevated temperature reduction of the surface layer may lead to the formation of a non-negligible amount of subsurface oxygen vacancies in ZnO; however, their presence and possible effect on the interaction between Cu and ZnO have not been evidenced so far.

In this report we reveal the details of the interaction between Cu NPs and the polar zinc-terminated ZnO(0001) surface. ZnO(0001) is more catalytically active than many other ZnO surfaces^7^,^8^, and hence studies of Cu on ZnO(0001) are of a considerable interest. In general, unreconstructed polar oxide surfaces possess high surface energies owing to a non-compensated surface dipole^9^,^10^. The polar surfaces of ZnO exhibit various stabilization mechanisms such as surface reconstructions (for example, triangular pits and islands^11^, surface-ion vacancies^12^, adsorption of foreign species such as, for example, hydrogen^13^ or other mechanisms constituting a change in surface composition^13^,^14^,^15^,^16^,^17^). The higher surface free energy of polar surfaces enables metals to bind stronger to such surfaces, which may cause the metal to wet the polar surface if the process is thermodynamically favourable and not kinetically hindered^18^,^19^,^20^. Wetting has been predicted theoretically, for example, in the case of Cu/MgO(111) (ref. 21) and Pd/MgO(111) (refs 10, 22). The theoretical predictions and experiment agree well in the case of Pd grown on an FeO(111) ultra-thin film^23^. In that case complete wetting was observed for Pd 3D nanoclusters grown on the FeO(111) thin film and it was concluded that the wetting at high temperature was driven by the polar nature of the FeO(111) film^23^. In our present study we unequivocally demonstrate that a 3D to 2D transition takes place in the Cu/ZnO(0001) system because of defect-related changes of the ZnO(0001) surface structure.

The growth of Cu on the polar ZnO(0001) surface^24^,^25^,^26^, as well as thermally induced effects^27^,^28^,^29^,^30^,^31^,^32^,^33^ have been the subject of extensive debate and have also been intensively investigated theoretically^34^,^35^,^36^. Previous investigations on Cu/ZnO(0001) (refs 31, 33) left several important questions about the thermally induced behaviour of this system, such as disappearance of 3D-like Cu NPs^31^, Cu-induced changes of the ZnO surface structure^31^ and Cu–Zn alloying^37^,^38^ upon annealing, unanswered. Part of this debate originates from the incomplete understanding of the structure of the ZnO(0001) surface itself.

By means of detailed scanning tunnelling microscopy (STM) and x-ray photoelectron spectroscopy (XPS) experiments combined with density functional theory (DFT) calculations we shed light on the structural and electronic interaction between Cu and the defects in the surface and subsurface region of ZnO(0001). Surprisingly, we observe that heating of the Cu/ZnO(0001) sample does not lead to the expected continuous coarsening of the Cu NPs, but to a fine atomic layer dispersion (wetting) of the initial 3D Cu NPs over the ZnO(0001) surface. This unusual phenomenon is explained by an increase in the Cu adhesion energy as a result of structural transformations on the ZnO(0001) surface, which is induced by the surface-directed migration of positively charged subsurface defects.

We propose a mechanism in which Cu deposited on the ZnO(0001) surface electrostatically interacts with ionized subsurface defects in ZnO within the depletion region upon the formation of a Cu/ZnO Schottky-like contact. The electric field generated in the depletion region consequently leads to the surface-directed drift of those defects upon annealing. The defects emerge at the surface, changing its structure, which is directly visible in STM images, causing a transition of 3D Cu NPs into a finely dispersed 2D layer. This mechanism implies that control over the subsurface and bulk defect concentrations accompanied by thermal treatment, can be used to change the structure and properties of metal-oxide interfaces. We believe that these results provide an important key to understanding of the dynamic behaviour of Cu/ZnO catalysts, as well as an interesting perspective on the laminar growth of seamless metal-oxide contacts based on ZnO.

## Results

### The ZnO(0001) surface

Although a number of different surface reconstructions on the ZnO(0001) surface were revealed by low-energy electron diffraction (see ref. 16 and references therein), the structure was never, to the best of our knowledge, resolved atomically by STM, possibly because of the relatively poor conductivity of ZnO (refs 39, 40). The most commonly observed morphology of ZnO(0001) consists of a large number of variable-size triangular terraces (presumably (1 × 1) terminated^11^) and a significantly smaller number of triangular pits with O-terminated step edges as first reported by Dulub *et al*.^11^,^41^. The triangular terraces and pits emerge under normal UHV surface-preparation conditions and help to reduce the surface polarity by increasing the O:Zn ratio. In contrast, high-temperature annealing leads to a flatter surface with every third surface Zn ‘row' missing^12^, where the partial loss of surface Zn atoms reduces the surface polarity.

**Figure 1:**
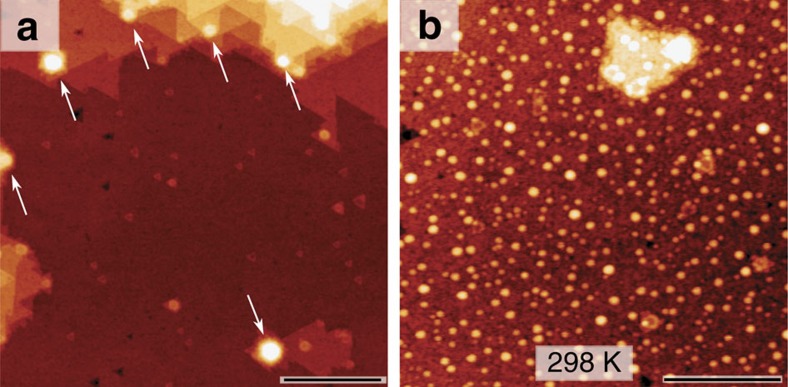
Deposition of Cu on ZnO(0001) at RT. STM topography images of the ZnO(0001) surface (**a**) before and (**b**) after the deposition of 0.3 ML of Cu at room temperature. Scale bar, 50 nm (**a**) and 30 nm (**b**). The arrows in **a** indicate features that result from partial decomposition of the ZnO(0001) surface during the preparation at high temperature (780 K). The STM images have been recorded in constant-current mode at ∼2 V of sample bias and 90–120 pA as a tunneling current set point.

The STM image in Fig. 1a shows the initial state of the bare ZnO(0001) surface used in our experiment before Cu deposition. On a large scale, this surface consists of large, flat terraces with a negligible amount of triangular pits and islands. This flat ZnO(0001) morphology can be achieved after a flash annealing at ∼780 K. Mass-spectrometry performed in front of the crystal (see Supplementary Fig. 1 and Supplementary Methods) revealed that high-temperature annealing results in the emission of a small amount of Zn, as well as some O_2_ and H_2_. On the microscopic scale the surface appears disordered with randomly placed dark depressions reminiscent of atom vacancies, but after a prolonged annealing at 780 K plus post-annealing at 760 K we observe ordered rows at the surface, like those reported earlier by Torbrügge *et al*.^12^ and interpreted as a reconstruction with missing Zn rows. The bare ZnO(0001) surface used in the experiments described below is therefore primarily stabilized by distributed surface Zn vacancies, with a negligibly small contribution of triangular islands and pits (that dominate surfaces prepared at lower temperatures^11^). This implies that Zn vacancies are present in the surface topmost layer with an effective coverage close to or slightly below ∼0.3 ML, in accordance with the stabilization criterion for this polar surface^9^.

### Thermally induced changes of the Cu NP morphology

One can expect that the growth mode and the properties of Cu on ZnO(0001) are strongly connected to the structure of the ZnO(0001) surface^34^,^36^ itself. The early studies showed that Cu grows on ZnO(0001) in the form of 2D islands up to ∼0.33 ML and continues to grow in the form of 3D-like islands above that coverage^24^, whereas recent STM studies^26^,^27^ showed that the change of the growth mode occurs below 0.05 ML. It was suggested that the variation in the growth regime was due to the difference in the ZnO(0001) surface-preparation conditions and the number of surface defects, which served as nucleation sites.

**Figure 2:**
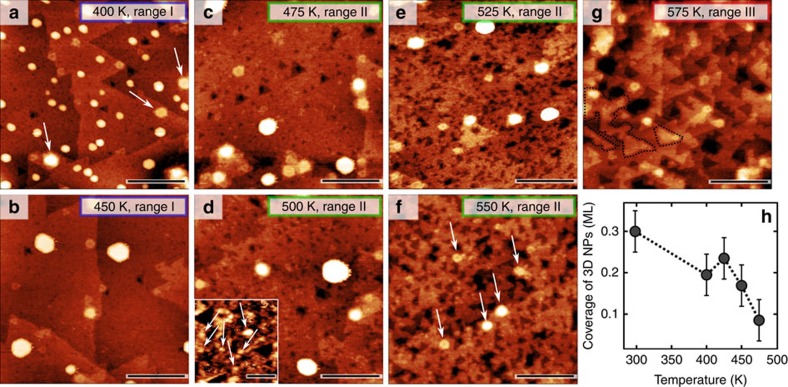
Effect of the annealing temperature on surface morphology. (**a**–**g**) The STM images acquired at RT after cyclic annealing of a Cu/ZnO(0001) sample in the 400–575 K range. Scale bar, 30 nm (for all of the images); 5 nm (for the inset in **d**); (**h**) The apparent coverage corresponding to the large 3D Cu nanoparticles versus temperature of annealing as estimated using 400 × 400 nm^2^ STM images.

**Figure 3:**
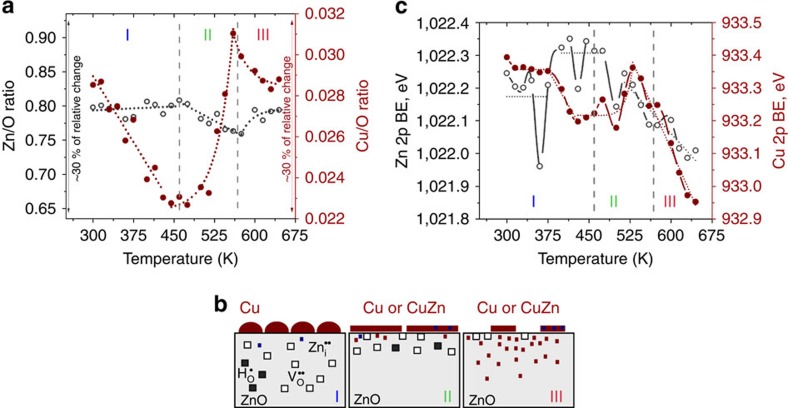
Effect of the annealing temperature on the XPS signal ratios and model representation of the thermally induced changes at the surface. (**a**) The Zn 2p (open symbols) and Cu 2p (filled symbols) corrected integral intensities referred to O 1 s intensity derived from the respective XPS spectra recorded at RT after subjecting the Cu/ZnO(0001) sample to cyclic annealing. (**b**) A schematic representation of the processes occuring in the three temperature regimes at the Cu/ZnO(0001) interface upon annealing. (**c**) The corresponding Zn 2p (open symbols) and Cu 2p (filled symbols) binding energies.

Figure 1b shows that after the room temperature (RT) deposition of 0.3 ML of Cu, there is a mix of 1–4 ML high 2D- and 3D-like clusters with diameters in the range of 1–3 nm, as estimated from their full-width at half maximum of the STM profiles, which is consistent with earlier STM studies by Koplitz *et al*.^26^. To investigate cluster coarsening and thermal effects, we performed a sequential series of STM and XPS measurements at RT after the samples had been subjected to annealing between 300–650 K. The annealing cycles consisted of a 1 K s^−1^ linear increase from RT, 5 min of annealing at constant temperature (T_set_) and a 1 K s^−1^ linear decrease down to RT. The STM data (Fig. 2a–g) and the XPS elemental ratios (Fig. 3a) correlate very well with each other and are consistent with coarsening of the Cu NPs between 300–465 K (range I). The initial 3D growth mode and the coarsening of the Cu NPs in range I indicate that the morphology of Cu in this range is determined by kinetic rather than thermodynamic factors^18^ and that, thermodynamically, Cu prefers not to wet the surface. However, the experimental results reveal a different trend from 465–560 K (range II) consistent with the transformation of 3D Cu NPs into a 2D layer of finely dispersed Cu and finally a loss of Cu between 560–650 K (range III).

In range I (Fig. 2a,b), the coarsening of the Cu NPs occurs presumably via a combination of Ostwald ripening and particle-coalescence mechanisms (in agreement with a previous study^28^). XPS is sensitive only to the few topmost layers of Cu, so coarsening of the 3D Cu NPs in this range should lead to an increased amount of bulk Cu which is attenuated in XPS (otherwise stated, there should be a decrease of the surface-to-volume ratio of Cu that would lead to a decrease of the Cu 2p integral intensity). This is fully confirmed by XPS (Fig. 3a), where the Cu/O ratio decreased with increasing temperature whereas the Zn/O ratio practically remained constant (increase <1%). However, starting from ∼450 K the NPs began to decrease in size as seen by STM. To quantitatively estimate the effective coverage of 3D Cu NPs we analysed the volume of such particles as obtained from the 400 × 400 nm^2^ STM scans recorded at different temperatures; the results are presented in Fig. 2h. The coverage of the large 3D NPs visible in STM dropped from ∼0.3 ML (1 ML is defined as the 2D packing density of copper ≅1.77 × 10^15^ atoms per cm^2^) at 300 K (range I) to ∼0.1 ML at 475 K (beginning of range II) and no 3D Cu NPs remained on the surface above 550 K (Fig. 2f, end of range II). A careful analysis of scans up to 600 × 600 nm^2^ from several random positions also did not reveal any 3D NPs above 550 K, which is consistent with the earlier study by Batyrev *et al*.^31^ Note that the bright features marked by arrows in Fig. 2f are not Cu NPs, but ZnO surface features, which are also found on the bare ZnO(0001) surface after annealing at 780 K (marked by arrows in Fig. 1a, see also Supplementary Note 1). The disappearance of the 3D Cu NPs owing to desorption is excluded, as TPD measurements showed no desorption of Cu. Instead, as the size of the big 3D NPs began to decrease above 450 K, the STM images revealed a number of tiny bright protrusions with the apparent height of 1–2 Å above the ZnO surface and ∼8 Å in lateral dimensions, which we ascribe to a layer of highly dispersed 2D Cu (or possibly Cu–Zn) nanoclusters (see inset in Fig. 2d).

The STM images reveal that the number of these small 2D nanoclusters increased with temperature, suggesting that Cu gradually transforms from large, coarsened 3D NPs found in range I into the finely dispersed 2D clusters found in range II, that is, consistent with the formation of a wetting layer. Evidence for this is provided by XPS: Fig. 3a shows that the decreasing trend in the Cu/O ratio from range I is now replaced in range II by a function that is exponentially increasing with temperature, whereas the Zn/O ratio decreases linearly. Such an increase of the Cu/O ratio, as well as of the Cu/Zn ratio (see also Supplementary Note 2 and Supplementary Fig. 2) is only consistent with the formation of a dispersed Cu overlayer on top of ZnO (that is, 3D Cu NPs transform into a 2D layer).

Upon annealing at temperatures above 560 K (range III), the 2D clusters developed into islands (a few of them marked by dotted lines in Fig. 2g). The Cu/O ratio (Fig. 3a) sharply decreased, indicating that Cu started to diffuse^42^,^43^ into the ZnO. The Zn/O ratio increased somewhat, presumably owing to Cu–Zn alloying^37^,^38^ at the surface and Cu bulk in-diffusion. The whole process is schematically shown in Fig. 3b.

### Cu-induced changes of the ZnO(0001) morphology

Above, we have focused on the Cu morphology, but importantly, as seen in Fig. 2a–g the ZnO surface morphology itself was also strongly affected by the thermally induced interaction with Cu. In range II, after annealing of the Cu/ZnO(0001) samples above 475 K, the initially very flat surface transformed into a rougher surface exposing single ZnO-bilayer-deep equilateral triangular pits (Fig. 2c–f) with an apparent depth of 2.9±0.3 Å. Sometimes larger pits (≅10–20%) with smaller triangular pits inside occur (like those initially reported by Dulub *et al*.^41^ on a clean UHV-prepared ZnO(0001)). It is important to note that the temperature range where we observed the appearance of the triangular pits (range II, 475–550 K) was significantly lower than the annealing temperature used for the ZnO(0001) surface preparation.

The lateral size, as well as the depth of the pits increase gradually during further cycles of annealing. The projected area occupied by such triangular pits expressed in terms of coverage corresponds to ∼0.27 ML at 525 K. This value is almost in 1:1 correspondence with the expected ∼0.3 ML coverage of the surface Zn vacancies on the bare ZnO(0001) surface, which strongly supports that the bilayer-deep triangular pits arise as a consequence of the coalescence of Zn and O vacancies at the surface. Batyrev *et al*.^31^ previously reported that such triangular pits possibly result from the migration of Zn and/or ZnO_*x*_ species on top of the 3D Cu particles, but we were unable to find any evidence for such mechanisms in our samples. The top facets of all of the Cu 3D NPs found in the range between 450 and 525 K were atomically flat and no signs of Zn or ZnO migration onto Cu were found (in contrast to, for example, the case of Pt/TiO_2_(110) (ref. 44), where TiO_*x*_ occurred on the top facets of Pt NPs). We also did not observe any desorption of oxygen or zinc in range II and III, which could lead to the emergence of the triangular pits. Thus, we conclude that the pits are formed as a result of Cu-induced rearrangement of the existing defects in the surface and subsurface layers which will be discussed below.

### Defects and charge transfer between Cu and ZnO(0001)

To investigate the appearance of the triangular pits we consider possible point defects in ZnO, as well as mechanisms which could lead to their migration towards the surface and coalescence. Among a number of native point defects which are known to be abundant in ZnO (refs 45, 46) the most likely candidates that may account for the appearance of hollow sites like the triangular pits seen in temperature range II are oxygen vacancies (V_O_, also called F-centres), substitutional hydrogen trapped on oxygen vacant sites (H_O_), and Zn vacancies (V_Zn_). The presence of increased concentrations of defects in the subsurface region of ZnO polar surfaces has been revealed experimentally in recent studies on the model Schottky diodes by depth-resolved cathodoluminescence^47^. It has also been concluded that the subsurface defects in ZnO are electrically active^47^ and tend to redistribute upon interaction with various metallic contacts placed on top of ZnO polar surfaces^48^.

During preparation we observed a significant emission of oxygen from the clean ZnO(0001) surface upon annealing in UHV above 760 K (see Supplementary Fig. 1), which indicates that such annealing leads to a partial reduction of the crystal and formation of oxygen vacancies. Taking into account that the Fermi level in our case lies very close to the bottom of the conduction band (discussed below) and that the residual partial pressure of oxygen during the experiments was below 1 × 10^−12^ mbar (detection limit), the formation energy of oxygen vacancies is expected to be rather high (3.72 eV (ref. 45)). Therefore, the annealing at ∼800 K alone cannot be responsible for the formation of oxygen vacancies and we speculate that the reduction process is enhanced by Ar^+^ sputtering used for surface cleaning in our experiments, similar to what was earlier observed on other oxides (for example, TiO_2_ (ref. 19)) and is possibly facilitated owing to the presence of hydrogen in the subsurface layer (see Supplementary Note 3).

**Figure 4:**
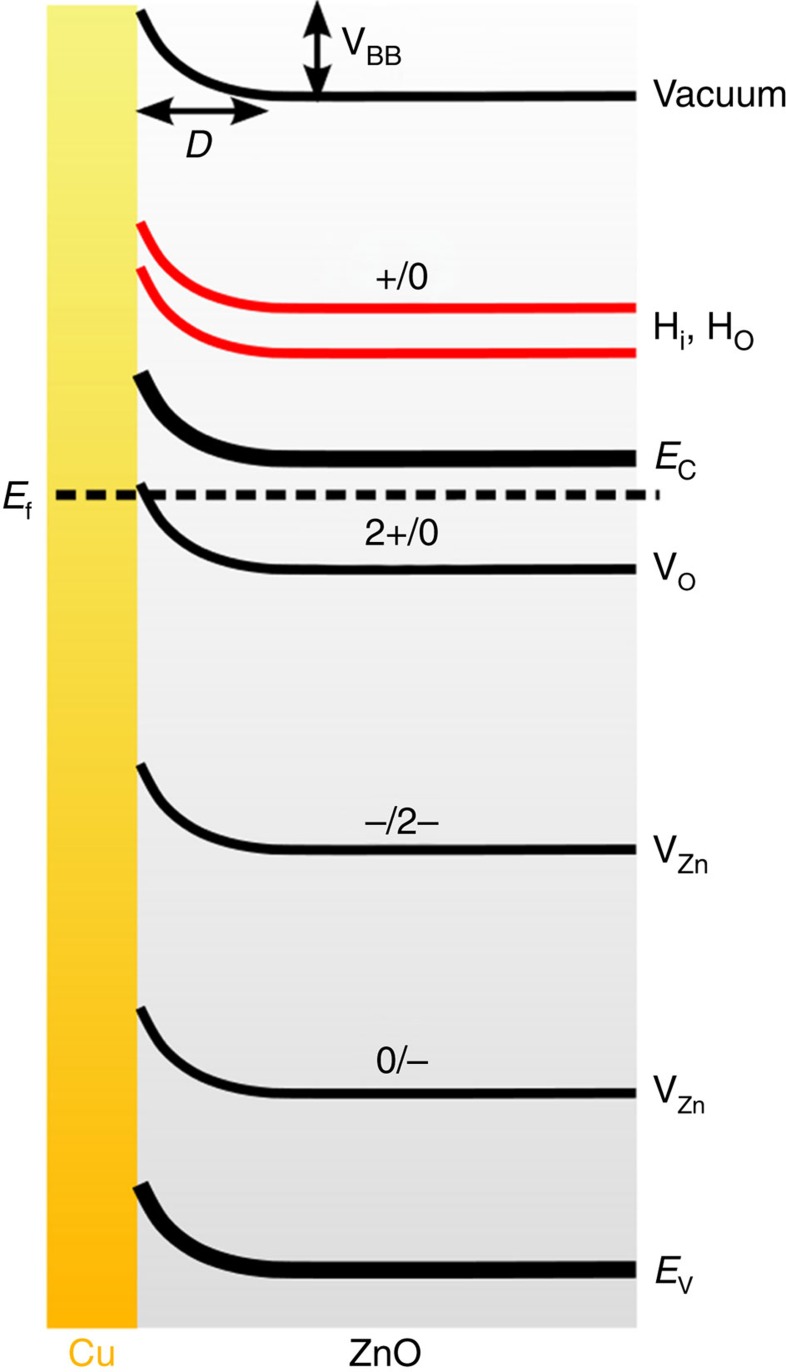
Band-bending at the surface and ionization levels of several considered subsurface defects. A schematic representation of the band-bending at the interface between the Cu NPs and ZnO(0001). The positions of charge-transition levels of some defects within the band gap of ZnO are qualitatively (not to scale) indicated (from refs 45, 50) by solid lines; (−) and (+) indicate the effective charges of defects (that is, 2− and 2+ corresponds to 
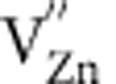
 and 
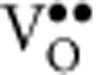
, respectively). The lines for H_i_, H_O_ (red) are tentative as we do not know the exact position of these charge-transition levels. The ionization of the oxygen vacancies close to the interface can be facilitated as the corresponding transition level for this defect may appear above the equilibrated Fermi level. The estimated amount of the band-bending V_*BB*_ ∼0.4 eV and the corresponding width of the depletion region D ∼8.9 nm, respectively (see Supplementary Note 4).

The above-mentioned defects in the surface layer become ionized either as a result of thermal ionization or, importantly, upon Cu deposition owing to the equilibration of the Fermi levels between Cu and ZnO (formation of a Schottky-like contact^20^,^49^, see schematics in Fig. 4). As a result of such ionization the defects can attain positive or negative effective charges. The defect charge-transition levels obtained from previous studies are schematically shown in Fig. 4 (refs 45, 50).

Since the driving force for the defect drift and rearrangement can be of electrostatic origin^20^, it is important to estimate the direction and the amount of charge transfer at the interface. Yoshihara *et al*.^24^ established that these quantities, for Cu at sub-monolayer coverage on ZnO(0001), depend on the Cu coverage and the ZnO(0001) preparation procedure, such that the direction of the electric field in the subsurface layer could not simply be predicted from consideration of Cu and ZnO electronic properties themselves. Therefore, we evaluated the direction of the charge transfer between the Cu NPs and ZnO(0001) substrate and the amount of Cu-induced band-bending by applying the method developed by Chambers *et al*.^51^ to the analysis of the leading edge of the O 2p XPS peak (see Supplementary Note 4). The same approach was recently applied for the investigation of the charge transfer on the water- and hydrogen-treated bare ZnO surfaces^52^. The bare surface in our experiments demonstrated slight upward band-bending (0.05 eV). The deposition of Cu on the surface led to a positive band-bending increase of ∼0.4 eV as shown in Fig. 4 (see also Supplementary Notes 4 and 8 and Supplementary Figures 3 and 5), which is consistent with a negative charge transfer to the Cu NPs.

Additional confirmation about the charge transfer direction can also be reached from the analysis of the Cu 2p and Zn 2p binding energy (BE) shifts^53^. The correlation between the Cu/O and Zn/O ratios (Fig. 3a) and the Cu 2p and Zn 2p BEs (Fig. 3c) indicate that Cu gradually transforms from 3D NPs to a 2D layer starting from the middle of range I and continuing until a complete transformation is achieved at the end of range II. The gradual transformation into a 2D layer leads to an increased interfacial area between Cu and ZnO, which progressively depletes the ZnO surface region as more negative charge is transferred to Cu. This is seen in the second half of range I, where the Zn 2p BE increases relative to its initial value, whereas the Cu 2p BE decreases.

**Figure 5:**
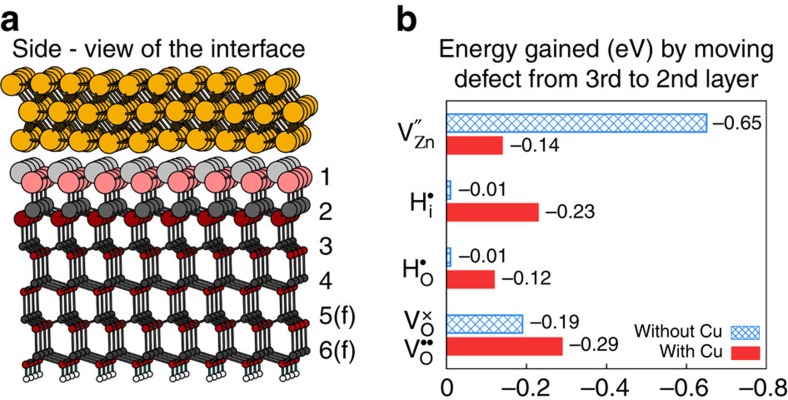
Energy gained via the surface-directed drift of charged subsurface defects. (**a**) Side-view of 3 ML Cu(111)/ZnO(0001) surface model (the surface-layer Zn and O are larger than subsurface Zn and O). The bilayers are numbered and the suffix ‘f' indicates that the corresponding atoms were kept fixed in the geometry optimizations. (**b**) Energy gained by moving subsurface defects from the third layer to the second layer (towards the surface/interface) both with and without 3 ML-adsorbed Cu metal.

Our first-principles calculations confirm the presence of negatively charged Cu adsorbates on ZnO(0001) as well. The Cu layer nearest to the interface for a 3-ML thick Cu metal overlayer (Fig. 5a) on the ideal ZnO(0001) surface obtains an average Bader charge of −0.05 a.u. per Cu atom. Such a Cu-induced charge separation has an important consequence: it creates an electric field, which acts on all of the effectively charged defects in the subsurface region and may lead to the drift of positive defects towards the interface upon annealing if the thermal energy is sufficient to overcome the migration barriers. The direction of the electric field in the depletion region thus favors the migration of positively charged defects towards the surface.

It is important to note that the charge transfer could potentially be affected in a similar way by the formation of copper oxide, although one would not normally expect the oxidation of Cu on ZnO(0001) upon annealing in UHV^54^. The migration of oxygen interstitials is also unlikely to cause the oxidation even though the migration barrier for them is sufficiently low (∼1 eV) simply because the oxygen intersitials would be either negatively charged or neutral and their migration towards the negatively charged Cu would be unfavourable. To clarify this issue we analysed the Cu 2p XPS peaks and did not reveal any shake-up satellites characteristic for CuO on ZnO(0001) (ref. 54) in the whole range of relevant temperatures. We also performed analysis of the so-called modified Auger parameter (AP) to evaluate the possible presence of a Cu(I) phase in the Cu/ZnO(0001) system upon annealing, as it is not possible to unambiguously distinguish between Cu(0) and Cu(I) based solely on the Cu 2p BE shifts. The AP helps to cancel out the contribution owing to surface charging and is defined for Cu as AP=KE(Cu LMM)+BE(Cu 2p 3/2), where KE and BE stand for kinetic and binding energy, respectively. In our case AP=1,856.6±0.4 eV and stays virtually constant for the whole range of measured temperatures; this value is neither consistent with the one expected for Cu_2_O (1,849±0.6 eV) nor with the one expected for Cu metal (1,851.20±0.3 eV), thus leaving the question about the exact oxidation state of Cu open. It is interesting to note that a comparably high AP (up to 1,854.3 eV) was observed in case of Cu in zeolites^55^.

### Charging and migration of subsurface defects

Using DFT, we investigated the migration properties of several relevant subsurface defects with and without the presence of a 3 ML Cu metal overlayer; the interface (Cu(111)/ZnO(0001)) is shown from the side in Fig. 5a (for top-view see Supplementary Figure 4). The defects denoted in Fig. 5b were introduced in the second or third ZnO bilayer below the Cu/ZnO interface. In this way, we evaluated the thermodynamic tendency for defect migration from ‘deep' in the bulk towards the surface. The resulting charge of the defects became 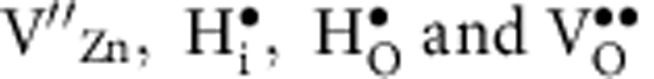 (to avoid confusion between the actual charges of the defects and their respective effective charges we use Kröger–Vink notation, see Supplementary Note 5). The oxygen vacancy V_O_ became ionized (
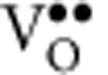
) only in the presence of the Cu overlayer; without the Cu, the high Fermi level made the oxygen vacancy neutral (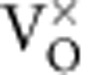). The band diagram in Fig. 4 indicates how the charge-transition level for V_O_ may follow the upwards band bending and reside above the Fermi level at the interface (see also Supplementary Note 6).

We find that it is always more favourable to place the defect in the second layer (nearer to the interface) than in the third layer because of the greater freedom to relax the defect structure, but depending on the defect charge, different trends are obtained depending on whether the Cu metal is present, see Fig. 5b. Without the Cu metal, the negative 
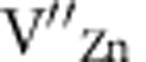
 is 0.64 eV more stable in the second layer than in the third, while this stabilization decreases to just 0.13 eV in the presence of the negatively charged Cu metal overlayer. In contrast, the energy gains for the positive 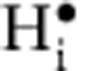 and 
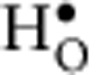
 species are very small without the Cu metal (0.01 eV), but these values increase to 0.22 and 0.12 eV in the presence of Cu. Similarly, the energy gain increases from 0.19 eV for 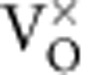 without Cu, to 0.29 eV for 
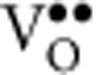
 in the presence of Cu. Thus, the migration of positive subsurface defects (specifically those associated with a missing bulk O: 
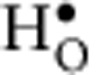
 and 
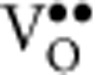
) towards the surface is enhanced in the presence of Cu, while the corresponding driving force for 
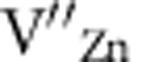
 is suppressed.

The migration barriers for 
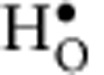
, 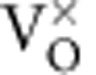 and 
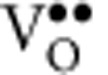
 in ZnO bulk have been calculated to be 2.5 eV (ref. 56), 2.4 eV (ref. 45) and 1.7 eV (ref. 45), respectively. Our STM data suggest that the migration barrier of the defect responsible for the emergence of the triangular pits (either 
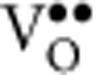
 or 
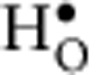
) is ∼1.5 eV (estimated using equation 9 from ref. 45). Thus, we speculate that in our case either a lowering of the migration barrier for 
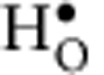
 owing to the presence of the surface dipole takes place or 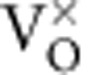 becomes doubly ionized 
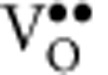
 as a result of the Cu-induced charge separation.

In conclusion, under experimental conditions, the shallow donors H_i_ and H_O_ are positively charged, and our calculations suggest that the oxygen vacancy V_O_ may also become positively charged within the Cu-induced space–charge region (Fig. 4). This makes it possible for these defects to drift towards the surface upon annealing.

### Binding of Cu to ZnO(0001)

**Figure 6:**
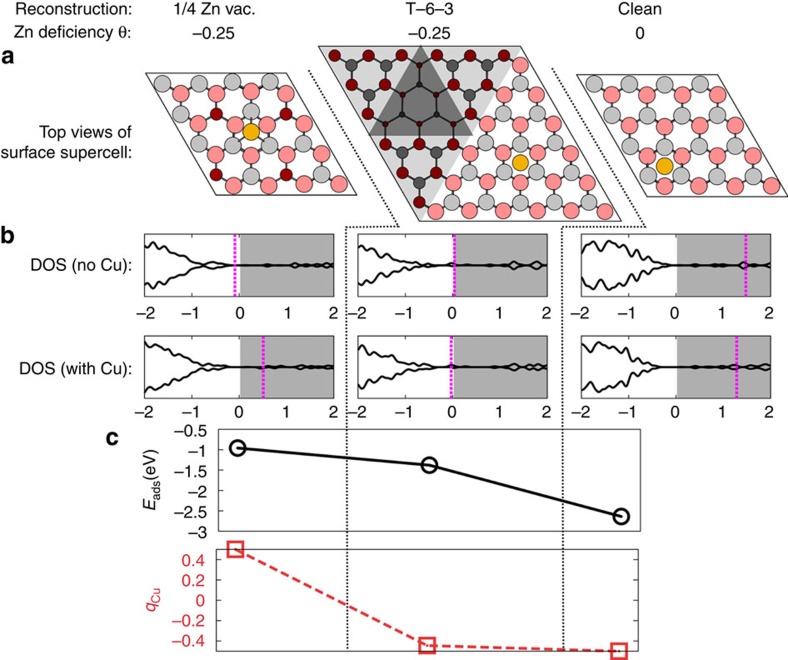
A correlation between the electronic properties, stucture and the adsorption strength of Cu at three differently reconstructed ZnO(0001) surfaces. (**a**) Top view of a Cu atom on the 1/4 Zn-vacancy reconstructed, the ‘T-6-3' reconstructed and the bulk-terminated ZnO(0001)-Zn surfaces. The Zn and O surface atoms (grey and red balls, respectively) are larger and brighter than the subsurface atoms; the pits have been shadowed. (**b**) Spin-polarized DOS for the respective reconstructed and bulk-terminated (0001) surfaces before and after Cu atom adsorption; the conduction band (shifted so that it starts at 0 eV) is shaded in grey and the Fermi level is denoted by a pink dashed line. The unit for the energy scale is eV. (**c**) The adsorption energy and the Bader charge q_Cu_ of the Cu atom adsorbed on the respectively reconstructed surfaces.

To finally understand what drives the increased wetting of Cu over the ZnO(0001) surface we investigated the adhesion of Cu on the initial Zn-deficient ZnO(0001) surface and several different surface reconstructions representative of the experiment. We will focus on the two most important reconstructions: a 1/4 Zn-vacancy reconstruction (in a 4 × 4 surface supercell) and a ‘T-6-3' reconstruction, which consists of a small triangular pit with a side length of 3 atoms inside a larger pit with a side length of 6 atoms (in a 6 × 6 surface supercell; nomenclature from ref. 36). Both of these reconstructions are shown in Fig. 6a and represent the initial surface (range I) and the surface after the emergence of the triangular pits (range II). Both have a surface Zn deficiency *θ*=−0.25 (refs 11, 57).

Figure 6b shows the calculated electronic density of states (DOS) in connection with the different reconstructions: for the 1/4 vacancy reconstruction, the conduction band (CB) is empty and the Fermi level lies within the band gap. In contrast, for the T-6-3 reconstruction, the Fermi level is at the CB minimum (which is similar to the Fermi level in actual, n-type ZnO), as a result of there being atoms at the corners of the triangular features, whose electrons are relatively high in energy and can be promoted into the ZnO CB.

A Cu atom was adsorbed at terrace-like regions on the different reconstructions. On the 1/4 Zn-vacancy reconstruction, Cu binds relatively weakly (*E*_ads_=−1.0 eV) and becomes positively charged, donating an electron to the ZnO CB (see DOS in Fig. 6b and Supplementary Note 6). In contrast, on the triangular pits (T-6-3) reconstruction, Cu binds more strongly (*E*_ads_=−1.4 eV) to the surface and becomes negatively charged, accepting an electron from the CB minimum. On an unreconstructed bulk-terminated ZnO(0001) surface, the Fermi level lies high in the CB and the adsorption is even stronger (*E*_ads_=−2.6 eV; much stronger Cu adsorption for a bulk-terminated as opposed to a Zn-deficient surface was also reported in refs 34, 36). Thus, provided that there are free electrons to accept (originating either from an ‘incomplete' surface reconstruction or from the presence of shallow donors), Cu will become negatively charged and bind more strongly to the ‘T-6-3' reconstructed ZnO surface. This is in agreement with the experimental observation of the gradual transformation of the 3D Cu NPs into a finely dispersed 2D layer of Cu upon the emergence of the high number of triangular pits in range II. In this way Cu also contributes to the stabilization of the ‘T-6-3' reconstructed surface, which emerges as a result of the drift of subsurface defects.

## Discussion

Despite the fact that the presence of subsurface defects has been highlighted in several recent model surface science studies on oxides^58^,^59^,^60^,^61^,^62^, there is surprisingly little information available about the role of such defects in metal-oxide interactions. The experimental studies of interactions between metallic adsorbates and subsurface defects in oxides are limited to a few cases where spectroscopic techniques were used^47^,^48^,^63^ and no information about the exact structure of the metal-oxide interface could be obtained. Our present study helps to fill this gap by clarifying the interaction mechanism between Cu and subsurface defects in the Cu/ZnO(0001) system.

Our results show that negative charge transfer from ZnO to Cu and the formation of a Schottky-like contact^49^,^20^ (schematically shown in Fig. 4) between these two materials cause the ionization of subsurface donors such as V_O_. Upon annealing in range II, H_O_ and V_O_ drift in the electric field created by the charge separation towards the surface and form triangular pits when they coalesce with the surface Zn vacancies. The emergence of the triangular pits modify the surface structure and thus leads to a change in the surface electronic and adsorption properties.

This helps us to explain the whole range of observed transformations of Cu on ZnO(0001) upon annealing. Initially, the ZnO(0001) surface contains many (up to 0.3 ML) surface Zn vacancies, that is, the surface is Zn deficient, which is necessary to remove the excess positive charge that gives rise to the polar instability. The adsorption of Cu on such a surface is relatively weak, and therefore Cu initially forms 3D NPs. After annealing in range II the ZnO(0001) surface undergoes a reconstruction (formation of triangular pits) owing to the emergence of the positively charged subsurface defects such as 
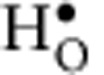
 and 
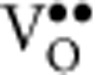
 attracted by the negatively charged Cu staying on the surface. The change of the ZnO(0001) surface structure leads to a stronger binding of Cu to the surface and ultimately to a fine dispersion (complete wetting, see also Supplementary Note 7) of Cu. The dispersed particles appearing in range II could also be because of Cu–Zn alloying, but such a scenario would require the migration of Zn interstitials as a driving force and can therefore be ruled out based on the observed XPS trends, which show no Zn enrichment in ranges I and II (see also Supplementary Discussion).

The proposed mechanism of Cu dispersion is confirmed by our STM, XPS and TPD results, and supported by the DFT calculations. The mechanism can be considered to be general and used for prediction and possibly modification of the wetting behaviour on other high surface energy (for example, polar) surfaces where the metal-adhesion energy can be modified as a result of the electrostatic interaction between charged-metallic adsorbates and ionized surface or subsurface defects. The charge transfer at the interface is one of the main prerequisites for the wetting to occur, and this will be fulfilled in most cases owing to the difference of the Fermi levels and electronegativities between a metal and an oxide. It is, however, important that the direction and the amount of charge transfer enable the drift of defects towards the surface and that the emergence of those defects leads to an increase in the oxide surface free energy.

Thus, the present study extends the knowledge about the role of subsurface defects in the metal-support interactions and explains the structural transformations in the Cu/ZnO(0001) system, which may be of relevance for other metal-semiconductor and metal-insulator systems as well.

## Methods

### Experimental details

The STM and XPS experiments were carried out in a UHV chamber with the base pressure below 1.5 × 10^−10^ mbar at RT using Zn-face EPI-polished ZnO(0001) crystals from MTI Corp. We used an Al K_α_ 250 W source with photon energy 1,486.7 eV for the XPS measurements. The measured integral intensities have been corrected by taking into account the inelastic mean free paths and the photoemission cross-sections for the electrons from Cu 2p, Zn 2p and O 1 s orbitals. The crystals were *in situ* cleaned by repeated cycles of Ar^+^ ion sputtering (1 keV, 15 min) and annealing (up to 780 K, 15 min) until a clean and well-structured surface could be observed in STM and no impurities, other than possibly some OH groups (a shoulder shifted ∼1.4 eV towards higher BEs with respect to the main O 1 s peak), were detected with XPS. Metallic Cu (0.3 ML) was deposited onto the ZnO(0001)-Zn sample held at RT from a copper rod (Goodfellow, 99.99+%) using an electron-beam evaporator (EGN4, Oxford Applied Research).

### Theoretical details

Spin-polarized DFT calculations were performed using the PBE^64^ density functional implemented in the VASP^65^,^66^,^67^,^68^ program. The plane-wave basis set had an energy cutoff of 500 eV and the electronic core-valence interactions were described by PAW^69^,^70^ potentials. A Gaussian broadening with *σ*=0.05 eV was applied to the electronic states.

The ZnO(0001)-Zn surface was modelled as a six bilayer-thick slab, where the O-terminated (bottom) side of the slab was passivated by pseudoatoms of valency 0.5 (see also Supplementary Methods), to prevent any interaction with this side when defects or adsorbates were introduced at the Zn-terminated side. Of the six bilayers, the top four (nearest to the Zn-terminated side) were geometry-optimized, while the bottom two and the pseudoatoms were kept fixed. Two different surface supercells were used: a 4 × 4 supercell, where the reciprocal space was sampled using a Γ-centred 3 × 3 × 1 k-point grid, and a 6 × 6 supercell, where only the Γ point was sampled. The vacuum gap in the surface normal direction was at least 13 Å and a dipole correction was applied.

## Additional information

**How to cite this article:** Beinik, I. *et al*. Enhanced wetting of Cu on ZnO by migration of subsurface oxygen vacancies. *Nat. Commun.* 6:8845 doi: 10.1038/ncomms9845 (2015).

## Supplementary Material

Supplementary Information Supplementary Figures 1-5, Supplementary Notes 1-8, Supplementary Discussion, Supplementary Methods and Supplementary References
