# Supplementary material for: Enhanced wetting of Cu on ZnO by migration of subsurface oxygen vacancies
Source: Nat Commun. 2015 Nov 16;6:8845. doi: 10.1038/ncomms9845 (PMC4660204; doi:10.1038/ncomms9845)
Supplement: Supplementary Information — Supplementary Figures 1-5, Supplementary Notes 1-8, Supplementary Discussion, Supplementary Methods and Supplementary References [file ncomms9845-s1.pdf]

Supplementary Figures

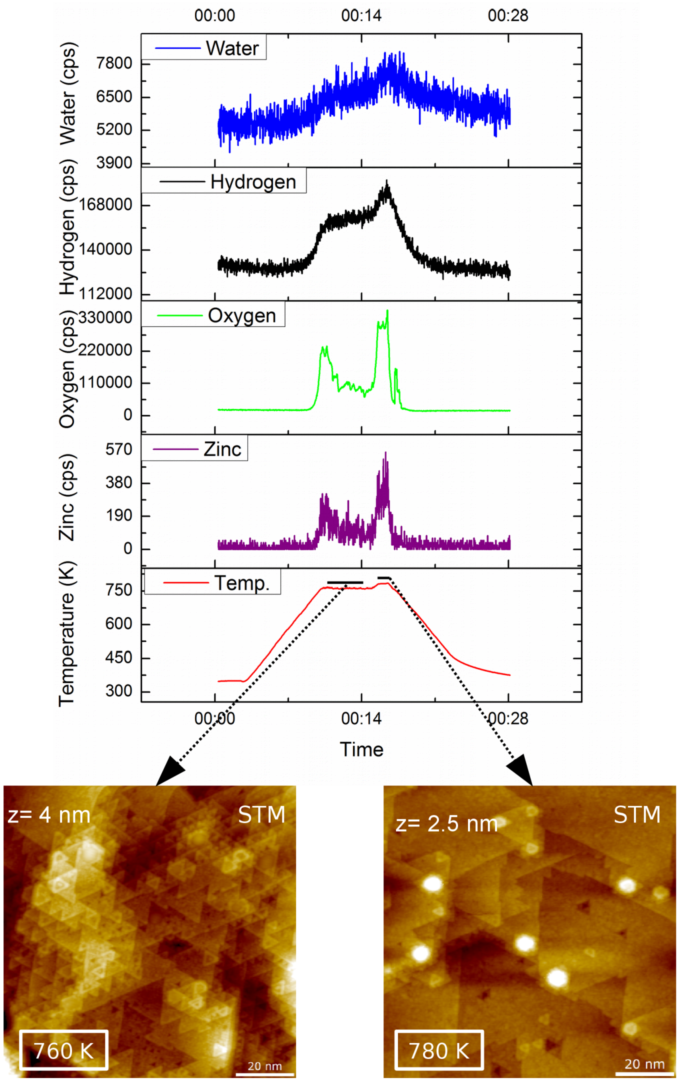

Supplementary Figure 1: Mass-spectroscopy measurements during sample preparation. The mass-spectrometry meaurements have been performed in a close proximity to the ZnO(0001) sample surface during the final cycle of the surface preparation (annealing).

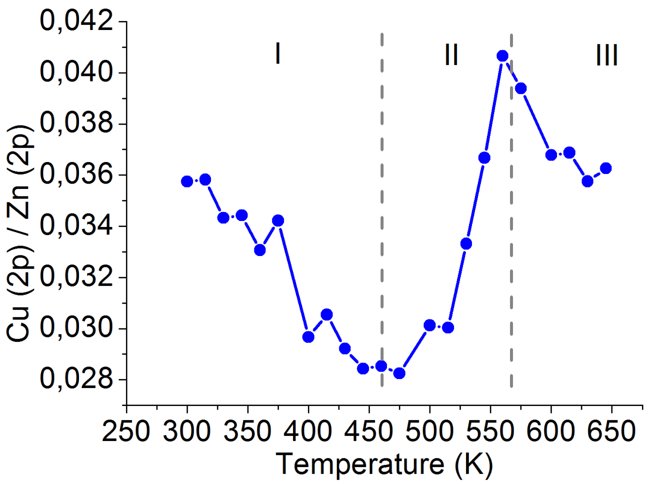

Supplementary Figure 2: Cu/Zn ratio vs. temperature. To complement the Zn/O and Cu/O ratios presented in the main text (Fig. 3(a)) we present the Cu/Zn 2p corrected integral intensities ratio as a function of the annealing temperature.

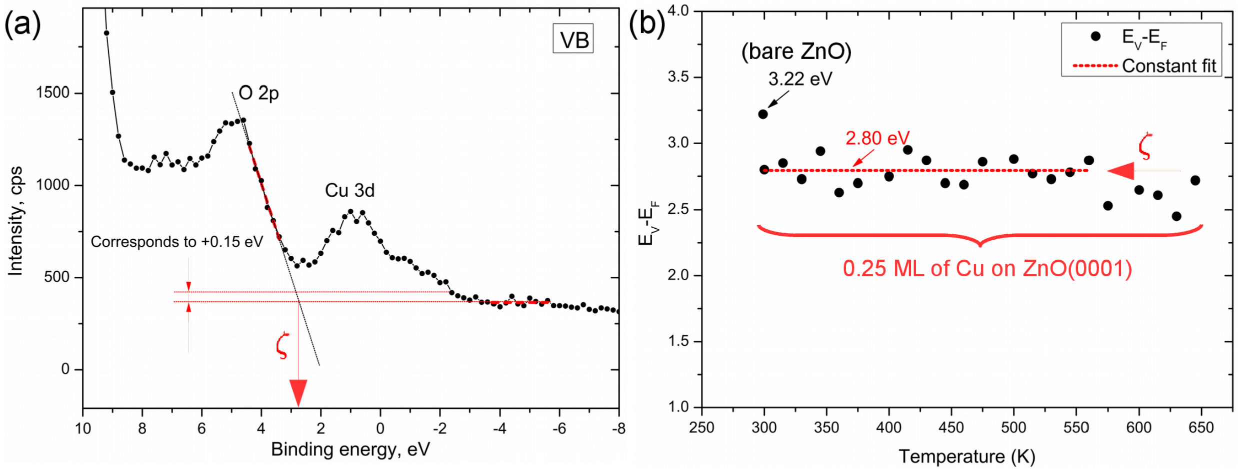

Supplementary Figure 3: Analysis of the ZnO(0001) surface band bending before and after the Cu deposition. (a) A characteristic valence band XPS spectrum of ZnO(0001) after the deposition of ≈0.3 ML of Cu. (b) A plot demonstrating the change of the $\zeta-$ parameter after the deposition of Cu and subsequent cycles of annealing.

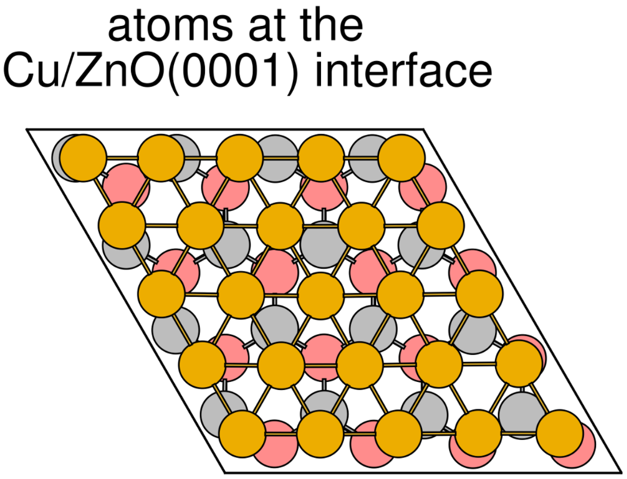

Supplementary Figure 4: Surface model used in DFT calculations. Top-view of only the interfacial atoms at the 3 ML Cu/ZnO(0001) interface in Figure 5(a) (main text). There are 5 × 5 Cu(111) surface unit cells on top of 4 × 4 ZnO(0001) surface unit cells.

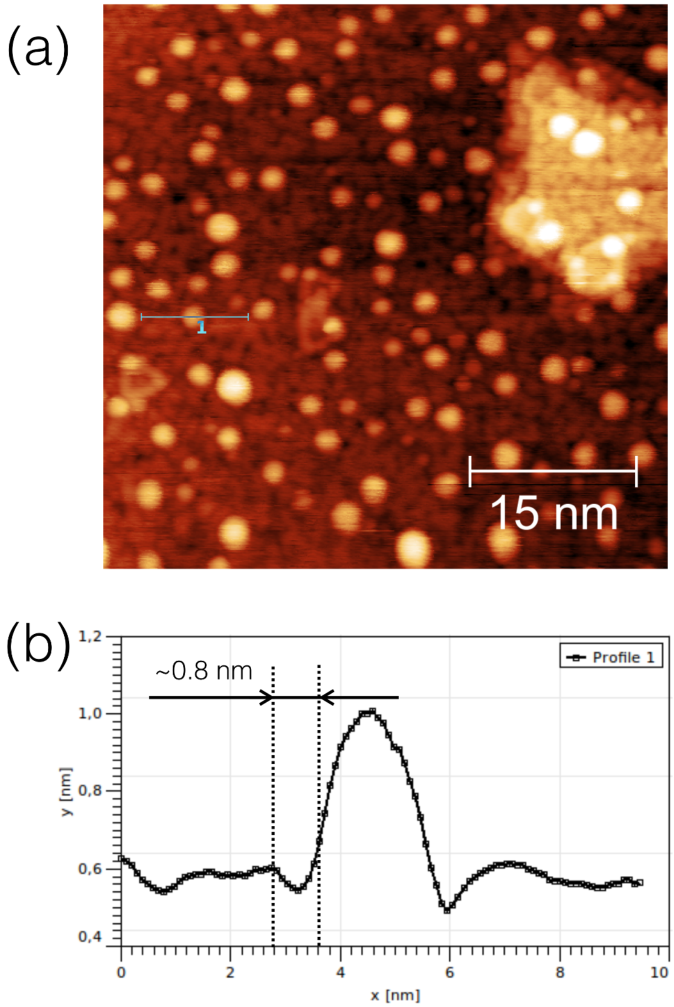

Supplementary Figure 5: Depletion region around Cu NPs. (a) An STM image of as-deposited Cu NPs on ZnO(0001). (b) A characteristic scan profile across one of the NPs along the blue solid line depicted in (a).

Supplementary Notes

Supplementary Note 1: Protruding features on a bare ZnO(0001) surface

The bright features marked by white arrows in Figure 1(a) and Figure 2 (a) and (f) of the main text appear as a result of partial decomposition of the surface at high temperature (780 K) and can serve as landmarks to distinguish between the original terraces formed at high temperatures and the overlayers, which could possibly appear after the Cu deposition and the thermal treatment at moderate temperatures (300 K–575 K).

Supplementary Note 2: XPS escape depth and elemental ratios

We would like to point out that the escape depth for 2p electrons in Cu at the given photon energy is ∼3.6 ML, which means that the signal from the interior atoms in the 2-4 ML high 3D Cu nanoparticles is attenuated compared to the situation where the Cu atoms are spread as a layer of adsorbates or film over the surface. Note that the XPS signal ratios are given instead of absolute intensities in order to cancel out a possible fluctuation of the X-Ray source intensity, although the absolute intensity trends are also fully consistent with the described sequence. The absolute integral intensity of the Cu 2p peak also increased to about the half of the initial value at the end of range II, while the Zn 2p intensity remained virtually constant after a pronounced drop at the end of range I. Moreover, the Cu/Zn ratio (see Supplementary Figure 2) increases from 0.028 at 475 K to 0.041 at 560 K (the end of range II), which is larger than the initial value of 0.036 after Cu deposition. The increase of the Cu/O ratio could in principle be affected by loss of oxygen. This process is however unlikely at the moderate temperature in range II and the TPD measurements confirmed that the Cu/O, Zn/O, and Cu/Zn ratios were not affected by a loss of species (e.g. Zn or O) from the surface since we did not observe desorption of any of the species within range II (O was emitted above 630 K, Zn above 715 K, and Cu above 750 K).

Supplementary Note 3: Hydrogen in the crystal

Our TPD and XPS measurements confirmed that the crystals we used for the experiments are chemically pure and did not contain any significant traces of impurities except for hydrogen. The TPD measurements (see Supplementary Figure 1) reveal a substantial amount of hydrogen desorbing from the as-grown ZnO crystal upon annealing. A deconvolution of the O 1s XPS peak recorded on the bare ZnO(0001) surface in accordance with the chemical shift assignments made in Ref. 1 also reveals a high concentration of hydroxyls in the surface layer. In fact, the effective coverage of OH equals to ∼2.1 ML for the bare surface before Cu deposition, which is clearly higher than can be accommodated on the surface. We consider this as an indication that a significant amount of hydrogen is residing in the subsurface layers.

In addition to regular oxygen vacancies and Zn vacancies, the substitutional hydrogen on an oxygen vacant site is an important and stable defect as it can be concluded from the analysis of the native point defects in ZnO[2,3] and our TPD and XPS experimental observations. The formation energy of H_O is significantly lower than for V_O (∼2.4–2.5 eV[4] vs. 3.72 eV[4] for the conditions of our experiments). This suggests that the presence of hydrogen may also facilitate the creation of oxygen vacancies (filled with hydrogen). It has recently been experimentally confirmed that omnipresent hydrogen in ZnO readily converts from interstitial hydrogen (H_i) into more stable H_O[4] upon annealing above ∼400 K[5]. Thus one can expect a high concentration of V_O and/or H_O in the subsurface region after the surface preparation by sputtering and annealing cycles. The presence of a relatively high amount of hydrogen in the crystal also accounts for its high conductivity. We found that a prolonged annealing of ZnO(0001) crystals in UHV above 900 K led to a significant emission of hydrogen, which was followed by a pronounced drop in the conductivity of the crystals (STM was no longer possible due to poor conductivity plus charging effects were clearly observed in XPS). As no other impurities except for hydrogen were found by XPS and V_O are rather deep donors (the level for a fully filled vacancy stays ∼1 eV below the conduction band minimum) the H_O is virtually the only feasible shallow donor.

Supplementary Note 4: Estimation of the Cu-induced band bending from the XPS data

In order to evaluate the direction of the charge transfer between the Cu NPs and ZnO(0001) substrate we estimated the amount of Cu-induced band bending by applying the method developed by Chambers et al.[6]. The same approach has recently been used for the investigation of the charge transfer on the water and hydrogen treated bare ZnO(0001) and ZnO(000$\bar{1}$) surfaces[1]. The amount of band bending was estimated as $V_{bb} = (E_g - \xi - \zeta)/e$, where E_g is the band gap (3.34 eV for ZnO) and $\xi = (k_BT/q) \cdot \ln(N_C/n)$ is the energy difference between the Fermi level and the bottom of the conduction band in ZnO ($\xi \approx 0.07\ eV$ for the effective density of conduction band states in ZnO $N_C = 2.94 \cdot 10^{18}$ cm^{-3} and the charge carrier concentration $n = 2 \cdot 10^{17}$ cm^{-3}). The $\zeta-$ parameter has been estimated experimentally from the projection of the intersection point of the extrapolated O 2p leading edge with the background level at 0 eV onto the x-axis (Supplementary Figure 3 (a)). The obtained values of the $\zeta-$ parameter are plotted vs. temperature of annealing in Supplementary Figure 3 (b). The amount of the band bending on the bare ZnO(0001) surface was obtained by averaging 5 subsequent measurements. In our studies we used hydrothermally grown crystals from MTI Corp. The crystals from this supplier are widely used and their electrical and optical properties have been quite thoroughly characterized[7]. It has been established that the charge carrier concentration in such crystals may change in a rather broad range depending on the hydrogen content starting from around 4.4$\times$10^{15} cm^{-3} for as-grown ZnO crystals to 7.1$\times$10^{17} cm^{-3} for intentionally hydrogen doped ones. We assume that the hydrogen concentration can be potentially very high as this is a parameter which is difficult to control and took the value 2$\times$10^{17} cm^{-3} for the estimation of the band bending, the same value as it was used in studies by Heinhold[1]. The corresponding Debye length estimated as $L_D = \sqrt{(\epsilon_{ZnO} \cdot \epsilon_0 \cdot k_BT/(q^2 \cdot N_d))}$ equals to approximately 7.8 nm for ϵ_{ZnO}=8.5. In case of the low carrier concentration reported for as-grown ZnO crystals from MTI Corp., the Debye length can potentially reach the value of approximately 50 nm.

The bare surface always demonstrated a slight upward band bending (0.05 eV, lies within the error bar). Since the background at 0 eV was unaccessible after the deposition of Cu we used an experimentally defined equivalent level between −4 eV and −6 eV, which

numerically coincided with the background level at 0 eV on the bare ZnO(0001) surface. A possible uncertainty in the determination of the ζ–parameter due to the fluctuations of the background level was determined to be within 0.15 eV (depicted in Supplementary Figure 3(a)). Thus, taking into account this uncertainty, we found that the amount of the positive band bending increased upon the copper deposition from ≈0.05 eV (bare ZnO) to ≈0.4 eV (with 0.3 ML of Cu), which was consistent with the negative charge transfer to the Cu NPs.

Supplementary Note 5: Kröger-Vink notation

An oxygen vacancy that contains two electrons is effectively neutral and denoted as $V_O^{\times}$, whereas an empty V_O is effectively positive and denoted as $V_O^{\bullet\bullet}$; the Zn vacancy V_{Zn}'' is effectively negative.

Supplementary Note 6: Reliability of the PBE functional

We note that the PBE method does not describe the charge transition level for V_O accurately. Therefore, the ionization of the oxygen vacancy with this method serves as a qualitative indication, rather than as an absolute prediction.

We would also like to note that the PBE functional underestimates the ZnO band gap; thus, in reality the adsorbed Cu atom need not necessarily act as a donor on the 1/4 vacancy reconstruction (although Cu was indeed found to become positively charged on ZnO(0001) at low coverage in Ref. 8).

Supplementary Note 7: The coverage of Cu chosen in our experiments

Here we would like to additionally point out that the 3D Cu NPs completely disappear from the surface and transform into a finely dispersed 2D layer due to their small coverage (0.3 ML), which was chosen to clearly demonstrate the effect. In case of higher coverages in the range from one to few MLs one can expect significant coarsening of 3D NPs accompanied

by their partial wetting of the ZnO(0001) and disappearance of some amount of Cu (proportional to the available surface area) as it converts into the finely dispersed phase. Some of the phenomena indeed have been observed[9] but not fully understood as was outlined in the introduction.

Supplementary Note 8: The effect of local Cu-induced band bending on the STM scan profiles

The depressions around Cu NPs, which arise due the local band bending, could be observed laterally by STM for most of the as-grown NPs. The observed width of such depressions is approximately 0.7-1.5 nm for 1-3 nm wide particles as determined from STM cross-section profiles (like the one depicted in Supplementary Figure 5 (b)), taking into account the tip broadening effect. This value corresponds to at least 2-3 double layers of ZnO, which means that the depletion layer would be sufficiently deep to overlap with the subsurface defects region. Although, it is also important to note that the width of the depressions is significantly smaller than the estimated width of the depletion layer. The width of the depletion layer for a nanoparticle on an oxide substrate can be obtained from the following equation: $\mathrm{V_{BB}} = \frac{\text{-eN}_\mathrm{d}}{\epsilon_{ZnO}\epsilon_0}\left[\frac{(D+\mathrm{r_m})^2}{2} - \frac{\mathrm{r_m^2}}{6} - \frac{(D+\mathrm{r_m})^3}{3r}\right]$, where D is the depletion layer width and $\mathrm{r_m}$ is the radius of a nanoparticle[10,11]. For a 1 nm particle, density of donor states $\mathrm{N}_d = 2\times10^{17}\ \mathrm{cm}^{-3}$, and $\mathrm{V}_{BB} = 0.4$ V (as estimated from the XPS O 2p leading edge analysis), the depletion layer width $D = 8.9$ nm, which is also very close to the Debye length estimated above.

There can be several reasons why the observed width of the depression region is smaller than the depletion layer width. However, the main reason is that the measured width of the depressions around NPs is a bias dependent quantity as it has been demonstrated in detail on a similar Cu/Pt(110) system by Carroll et al.[12]. Therefore, the charge depletion layer around Cu NPs could not be fully visualized in our apparent height empty state STM images.

Supplementary Discussion

The dispersed particles appearing in range II could also be due to CuZn alloying, but such a scenario would require the migration of Zn interstitials as a driving force and can therefore be ruled out based on the observed XPS trends, which show no Zn enrichment in ranges I and II. Furthermore, the theoretically predicted formation energy for Zn interstitials in n-type ZnO is very high (∼6 eV, Ref. 2), which should lead to low concentrations, in agreement with our experimental observations. In range III, however, Zn interstitials could form during Cu bulk in-diffusion, according to the substitutional mechanism proposed by Qiu et al.[13]. Since Zn interstitials are highly mobile[2], we tentatively conclude that the Zn/O ratio increase in range III is caused by two factors, namely Cu bulk in-diffusion and Cu-Zn alloying at the surface.

Supplementary Methods

Experimental procedures

The samples for the STM and XPS measurements have been prepared in a UHV chamber with the base pressure below 1.5×10^{-10} mbar. We used Zn-face EPI polished ZnO(0001) crystals from MTI Corp., which were in-situ cleaned by up to 20 times repeated cycles of Ar^+ ion sputtering at 1 keV (15 min) and annealing up to 780 K (15 min). We also performed mass-spectroscopy measurements in a close proximity to the surface during the final cycles of sample preparation. The temperature was monitored by a standard K-type thermocouple attached to one side of the sample and was simultaneously cross-checked by a pyrometer. The two temperature readings coincided only in a narrow range around 500 K but could be reproducibly linked by the empirically determined relation $T_{\text{pyro}}\,[\text{K}]=1.9\cdot T_{\text{tc}}-484.1\,[\text{K}]$, where T_{pyro} and T_{tc} are the temperatures measured by the pyrometer and the thermocouple, respectively. It can be seen in Supplementary Figure 1 that the emission of components such as hydrogen, oxygen and zinc are strongly dependent on the temperature of annealing. We found that the morphology and the flatness of the resulting surface could be correlated with the temperature of annealing.

Theoretical procedures

A 3 ML Cu metal overlayer with Cu(111) ∥ ZnO(0001) is shown in Figure 5(a) (main text). In the calculations with 3 ML Cu, the 4×4 supercell was used for ZnO, and 5×5 Cu(111) surface unit cells were used for Cu (see the "top view" of the interfacial atoms in Supplementary Figure 4). Because of the lattice mismatch (PBE-optimized Cu(111) surface unit cell $L = 2.567$ Å, PBE-optimized ZnO(0001) surface unit cell $L = 3.288$ Å), there is a small amount of strain in the Cu (2.5%, i.e., the Cu is elongated compared to bulk-like Cu) in the lateral directions. This kind of surface model has also previously been used to model Cu metal adsorbed on ZnO(0001)[14].

The V_{Zn}, H_i, H_O, and V_O defects were introduced in the second and third ZnO bilayers beneath the interface (labelled 2 and 3 in Figure 5(a) of the main text), in order to evaluate the thermodynamic tendency for defect migration towards the surface from "deep" in the bulk, both with and without 3 ML Cu adsorbed on the surface.

For the clean (unreconstructed) ZnO(0001) surface, the polarity gives rise to an electric field over the slab which drives $\frac{1}{2}$ electron per surface atom from the O-terminated side to the Zn-terminated side. The holes created at the O-terminated side are filled by electrons from the pseudoatoms, and the end result is that there are electrons which populate the conduction band at the Zn-terminated side (corresponding to the *surface metallization* stabilization mechanism). These electrons can be depleted from the conduction band if the surface stoichiometry at the Zn-terminated surface is changed through the removal of Zn ions or addition of O ions, or if species which accept electrons (e.g. Cu atoms) are adsorbed.

Supplementary References

[1] Heinhold, R., Williams, G. T., Cooil, S. P., Evans, D. A. & Allen, M. W. Influence of polarity and hydroxyl termination on the band bending at ZnO surfaces. *Phys. Rev. B* **88**, 235315 (2013).

[2] Janotti, A. & Van de Walle, C. G. Native point defects in ZnO. *Phys. Rev. B* **76**, 165202 (2007).

[3] Janotti, A. & Van de Walle, C. G. Fundamentals of zinc oxide as a semiconductor. *Rep. Prog. Phys.* **72**, 126501 (2009).

[4] Janotti, A. & Van de Walle, C. G. Hydrogen multicentre bonds. *Nat. Mater.* **6**, 44–47 (2007).

[5] Koch, S. G., Lavrov, E. V. & Weber, J. Interplay between interstitial and substitutional hydrogen donors in ZnO. *Phys. Rev. B* **89**, 235203 (2014).

[6] Chambers, S. A., Droubay, T., Kaspar, T. C. & Gutowski, M. Experimental determination of valence band maxima for $SrTiO_3$, TiO_2, and SrO and the associated valence band offsets with Si(001). *J. Vac. Sci. Technol. B* **22**, 2205–2215 (2004).

[7] Ton-That, C. *et al.* Shallow carrier traps in hydrothermal ZnO crystals. *New J. Phys.* **16**, 083040 (2014).

[8] Yoshihara, J., Campbell, J. M. & Campbell, C. T. Cu films on a Zn-terminated ZnO (0001) surface: structure and electronic properties. *Surf. Sci.* **406**, 235–245 (1998).

[9] Batyrev, E. D., Shiju, N. R. & Rothenberg, G. Exploring the Activated State of Cu/ZnO(0001)–Zn, a Model Catalyst for Methanol Synthesis. *J. Phys. Chem. C* **116**, 19335–19341 (2012).

[10] Ioannides, T. & Verykios, X. E. Charge Transfer in Metal Catalysts Supported on Doped TiO_2: A Theoretical Approach Based on Metal–Semiconductor Contact Theory. *J. Catal.* **161**, 560–569 (1996).

[11] Zhang, Z. & John T Yates, J. Band Bending in Semiconductors: Chemical and Physical Consequences at Surfaces and Interfaces. *Chem. Rev.* **112**, 5520–5551 (2012).

[12] Carroll, D. L., Wagner, M., Rühle, M. & Bonnell, D. A. Schottky-barrier formation at nanoscale metal-oxide interfaces. *Phys. Rev. B* **55**, 9792–9799 (1997).

[13] Qiu, H., Gallino, F., Di Valentin, C. & Wang, Y. Shallow Donor States Induced by In-Diffused

Cu in ZnO: A Combined HREELS and Hybrid DFT Study. *Phys. Rev. Lett.* **106**, 066401 (2011).

[14] Warschkow, O., Chuasiripattana, K., Lyle, M. J., Delley, B. & Stampfl, C. Cu/ZnO(0001) under oxidating and reducing conditions: A first-principles survey of surface structures. *Phys. Rev. B* **84**, 125311 (2011).
